# Quantifying the use of natural history collections

**DOI:** 10.3897/BDJ.12.e130811

**Published:** 2024-10-23

**Authors:** Max Caspers, Luc Willemse, Niels Raes, Erik Smets, Peter Schalk, Olaf Bánki, Gideon Gijswijt

**Affiliations:** 1 Naturalis Biodiversity Center, Leiden, Netherlands Naturalis Biodiversity Center Leiden Netherlands; 2 Catalogue of Life, Amsterdam, Netherlands Catalogue of Life Amsterdam Netherlands

**Keywords:** physical use, digital use, standards, quantification, parameters

## Abstract

Measuring the use of natural history collections is essential to understand their past and present impact on science, to underpin decisions about their management and to assist with deploying them optimally to address societal challenges. Using the vast natural history collections of Naturalis Biodiversity Center as an example, this paper assesses the significance and relevance of quantifying collection use. Four aspects are discussed: 1. standardisation, 2. relevance of having standardised metrics on collection use, 3. the level of detail and completeness of the information and 4. the interactions between digitisation of collections and physical collection use. Based on a set of transparent and objective parameters to describe collection use, it is proposed to further develop these into international standards.

## Introduction

Worldwide, thousands of repositories house natural history collections (NHC) that are essential for documenting and studying the biological diversity of our planet. The most extensive online global registry of these collections is provided by the Global Registry of Scientific Collections (GRSciColl) which is maintained by the Global Biodiversity Information Facility - GBIF (Hobern et al. 2020). Repositories are located in national institutes with millions of specimens kept in climate-controlled, often very large, warehouses, but much smaller privately-owned collections also contribute to the global knowledge base. A common characteristic amongst all repositories is that they aim to preserve and curate collections and make them accessible according to the FAIR principle for current and future use.

The biological and geological coverage of the global NHC is enormous and their diversity is as varied as the repositories that house them (Meineke et al. 2018). NHC vary in size, taxonomic focus, geographical origin, cultural history and methods to preserve objects. Not surprisingly, there is no consensus on a definition for NHC. We define an NHC as a collection that comprises a diverse range of physical specimens, including seed banks, botanical and zoological gardens, frozen or dried tissue collections and DNA banks, as well as geological objects: rocks, minerals and sediment cores of terrestrial and extraterrestrial origin. Libraries and archives are not considered NHCs in this paper.

The overall importance of NHC has been highlighted from various angles in numerous papers, editorials, book chapters etc. Bakker et al. (2020) illustrate the importance of NHC from the perspective of a ‘Global Museum’ where the relevance of museum collections is increasingly used in new ways that significantly expand their impact and relevance. A recent book published by the National Academies of Sciences, Engineering and Medicine (National Academies of Sciences, Engineering, and Medicine 2020) zooms in on the importance of NHC to advance species discovery, inspire innovation and inform about societal challenges such as contributing to education and lifelong learning and what is required to optimise their use now and in the future. NHC specimens capture basic biodiversity information (evidence) represented by their taxonomic name, place and time of collection, but also hold DNA that can be extracted and sequenced, represent traits that can be measured, hold historical information on their collector(s) and even include information about species interactions (e.g. pollen on an insect, fungus on a plant) and much more.

The importance of *mobilising* the information captured by NHC specimens through digitisation was recognised over twenty years ago with the establishment of the Global Biodiversity Information Facility (GBIF) as a common, free and open access portal to biodiversity data (CODATA 2020). This global portal unlocks close to 3 billion records of collection and observation data, a number roughly doubling every five years (GBIF metrics). Regional initiatives such as the Atlas of Living Australia (ALA), the Integrated Digital Biocollections (iDigBio) in the USA and the development of the Distributed System of Scientific Collections (DiSSCo) research infrastructure in Europe (Raes et al. 2020) aim to digitise and digitally open up all natural history collections under common access, curation, policies and practices. It is important to ensure that all data are easily Findable, Accessible, Interoperable and Reusable (FAIR principles) (Lannom et al. 2020). Some initiatives look at collections from an economic perspective and zoom in on the financial benefits of digitising collections and accelerating taxonomic discoveries in this realm, thus enhancing societal use (Davis 2021; Deloitte Access Economics 2021). The Catalogue of Life provides a continuously updated, open-access, authoritative global index of all known species names and synonyms facilitating interoperability between digital biodiversity resources, including the described digital collections.

NHCs are a *basic scientific infrastructure* as is evident from the steady flow of researchers visiting collections and the research papers based on NHC specimens. Digitisation promotes a fast growing use of natural history collections, underpins future collection policies and assists in meeting scientific and societal challenges (Johnson et al. 2023). However, coherent information detailing the use of NHCs is still scarce, heterogeneous and hard to access. Examples of published information on collection use include citations of datasets that are shared via GBIF (see, for instance, Hardy et al. (2023)) and more indirectly by specimen citations in taxonomic treatments. Research infrastructures such as GBIF, Biodiversity Literature Repository, Biodiversity Heritage Library and, for example, research projects such as BiCIKL and organisations such as Plazi aim to facilitate online access to specimen information in taxonomic treatments, for example, through services like Treatment bank which also includes statistical analyses.

Naturalis Biodiversity Center and its predecessors initially collected data on the use of the physical collections in a rather unstructured way, for example, through visitors' books, but in recent years this became more structured through the use of visitor and loan forms. Collection digitisation not only facilitates online access to collections, but also allows for a more detailed reporting and analysis of collection use. In this paper, we discuss four aspects of quantifying collection use: 1. relevance of having standardised metrics on collection use, 2. the level of detail and completeness of the information, 3. the challenges with standardising parameters and 4. the interactions between digitisation of collections, online publication and physical collection use. A generic set of parameters is proposed to quantify the scientific use of NHCs, with the aim to promote this as a global standard for measuring collection use.

## The Naturalis Biodiversity Center Collection

Naturalis Biodiversity Center was founded as the ‘Rijksmuseum van Natuurlijke Historie’ (RMNH) in 1820. During its 200 year history (Gijzen 1938, Brongersma 1978, Holthuis 1995, Erkens and Baas 2008, Reitsma 2012, Gassó-Miracle and Monquil-Broersen 2020, Schilthuizen and Vonk 2020), the size of the collection increased as a result of a series of mergers. The most recent one took place between 2011 and 2014 after the establishment of the current ‘Naturalis Biodiversity Center’ through the merger of Naturalis with the Leiden (L), Utrecht (U) and Wageningen (WAG) branches of the National Herbarium of the Netherlands (NHN) and the Zoological Museum Amsterdam (ZMA). The Naturalis Biodiversity Center has a collection of approximately 43 Million objects (Naturalis Bioportal) including botanical (acronyms L, U, WAG, ZMA), geological (acronym RGM) and zoological specimens (acronyms RMNH, ZMA). Table 1 presents an overview of subcollections present in Naturalis Biodiversity Center (reference date 2022) with the number of specimens held in the collection and those accessible online via GBIF.

**Figure 1.**
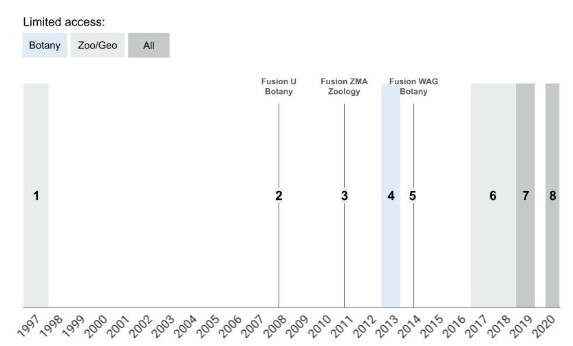
Events affecting collection access between 1997-2020 at Naturalis (incl. predecessors). Collection moves (M) and closures (C) in the period 1997-2020 at Naturalis and its predecessors: 1. Zoology/Geology - Rijksmuseum van Natuurlijke Historie, RMNH (M) 2. Botany - Utrecht Herbarium Utrecht, U (M) 3. Zoology - Zoölogisch Museum Amsterdam, ZMA (M) 4. Botany - Nationaal Herbarium Nederland, NHN (M) 5. Botany - Herbarium Vadense, WAG (M) 6. Zoology/Geology - Naturalis (C) 7. Zoology/Geology/Botany - Naturalis (M) 8. Zoology/Geology/Botany - Naturalis (C). In 1997 (event **1**), the zoological and geological collection of the former Rijksmuseum van Natuurlijke Historie had to be moved from its building in the centre of Leiden to the collection tower at the current Naturalis location on the outskirts of Leiden. In 2008 (event **2**), the Utrecht Herbarium (U), since 1999, part of the National Herbarium of the Netherlands moved to Leiden and stored in the same building as the Leiden Herbarium (L). In 2010, the former Naturalis merged with the National Herbarium Netherlands and the Zoological Museum Amsterdam into the Naturalis Biodiversity Center. This was followed in 2011 (event **3**) by the move of the collection of the Zoological Museum Amsterdam to Leiden and consequent fusion with the Naturalis collection. In 2013, the lease agreement for the building housing the botanical collections was not renewed and collections had to be moved to a temporary location at the eastern outskirts of Leiden (event **4**). The Herbarium Vadense Wageningen, part of the National Herbarium Netherlands, moved to a temporary location in Leiden in 2014 (event **5**) awaiting a move to the new Naturalis building. Due to construction activities for the new Naturalis building (2017-mid 2019), access to zoological and geological collections which were located in the middle of the actual building site were limited to 4 hours per month (event **6**). In 2019, all botanical collections were moved to the renovated Naturalis building. As a result, the botanical collection was closed by the end of 2018 in preparation for the move in 2019 (event **7**). The new Naturalis Biodiversity Center officially opened again on 31 August 2019 and business returned to normal. Unfortunately, this came to an abrupt end when COVID-19 reached Europe in 2020 - Q2 (event **8**).

To house the ever-expanding collection and to incorporate new technologies and insights on collection management and conservation, collections have been moved a number of times in the past, but more frequently between 1997 and 2020. Collection movements in this latter period severely limited physical access (Fig. 1) which affected collection use.

## Definition of collection use

The term “use” is defined as instances in which collection specimens are accessed for scientific purposes in ways that can be measured. The use of collections for education and exhibition has been excluded from this paper as we focused on facilitating research. The handling of specimens as part of preservation or collection management activities is also not considered as use in the context of this paper. The consultation of libraries and archives has also been excluded.

A distinction is made between digital and physical use. Digital use is defined as the downloading or citing of online data linked to specimens stored in the Naturalis Biodiversity Center. Browsing and undefined page visits were excluded as they lack essential information about the viewers (users) and their purpose. Physical use is defined as physical access to collection specimens (handling, annotating, studying, photographing) for scientific purposes. Four categories of physical collection use are distinguished:

1. on-site use by Naturalis Biodiversity Center staff (students, research staff, honourary staff),
2. on-site use by external visitors,
3. on-site use to meet with DNA or digitisation-on-demand requests and,
4. off-site use via outgoing loans.

Although use by Naturalis Biodiversity Center staff (1) and DNA or digitisation-on-demand requests (3) are amongst the most frequent use cases, structured qualitative and quantitative information on these use categories was not available and, thus, not included in this analysis. Standard administrative protocols at Naturalis Biodiversity Center record external visitors and outgoing loans only. Therefore, the metrics used in this paper to illustrate physical use of the Naturalis Biodiversity Center collection are related to visitors (2) and loans (4) only.

## Sources for visits and loans

Obtaining a combined historical overview of the use of the Naturalis Biodiversity Center collection proved challenging because of the heterogeneous nature of the information sources, in part due to the history of mergers. As botanical, zoological and geological collections used different protocols and digitisation started at different moments and at different speeds, the availability and consistency of the information differs. Here follows a brief summary of the sources that have been included or excluded from this paper.

**Visits**

External visits to the Naturalis Biodiversity Center collections have been recorded consistently since 2010. For the zoological and geological collections, this is the only period for which consistent data are available. The visitor registration for these subcollections prior to 2010 was carried out in a fragmentary manner. Visits are recorded at the level of larger subcollections like Aves (birds) or Mollusca (molluscs). Botanical collections, prior to the merger, had a practice of keeping visitors' books, of which only the Leiden book remains. Visitor books from the Utrecht, Wageningen and Amsterdam collections are missing. The Utrecht collection was housed in the same building as the Leiden collection after its move in 2008, so this collection is also registered in the Leiden visitors' book from that date. The Leiden botanical visitors' book was recently digitised and contains information on visitors to the collection from 1987 onwards. In 2013, this system was complemented with visits to all botanical collections in Leiden registered in Google Calendar. From 2014 onwards, only Google calendar has been used to register visitors of the botanical collections of Naturalis Biodiversity Center.

**Loans**

From the beginning of 1955, a paper administration was used by the predecessors of Naturalis to manage the outgoing loans from the zoological collections. A digital register was introduced in 1984, the year in which the geological collections of the former ‘Rijksmuseum van Geologie en Mineralogie’ (RGM) were moved to Naturalis. Only the still outstanding loans were entered into the system; hence, only the new loans from 1984 onwards are considered representative for the use of the zoological and geological collections from Leiden and have been analysed in this paper. The 1955-1984 zoological loan administration is available, but awaits digitisation. No information is available on loans of the geological collection prior to its move in 1984. Loans from the zoological collections of Wageningen and Amsterdam, after their move to Leiden, respectively in 2010 and 2011, are registered digitally in the system of Naturalis Biodiversity Center. Older loans from these institutions are available, but await digitisation.

In 1997, the botanical collection in Leiden started to register loans in the Brahms database system, followed by Wageningen in 1999 and Utrecht in 2000. These database files were merged when the physical collections moved to Leiden, in 2008 (Utrecht) and 2014 (Wageningen). The pre-1997 paper loan administrations of the Leiden, Wageningen and Utrecht Herbaria are also not yet digitised.

## Physical collection use

Data on physical and digital collection use of Naturalis Biodiversity Center were analysed and are presented here as: 1) general patterns and trends and 2) comparisons between subcollections.

**General patterns and trends**

**Figure 2.**
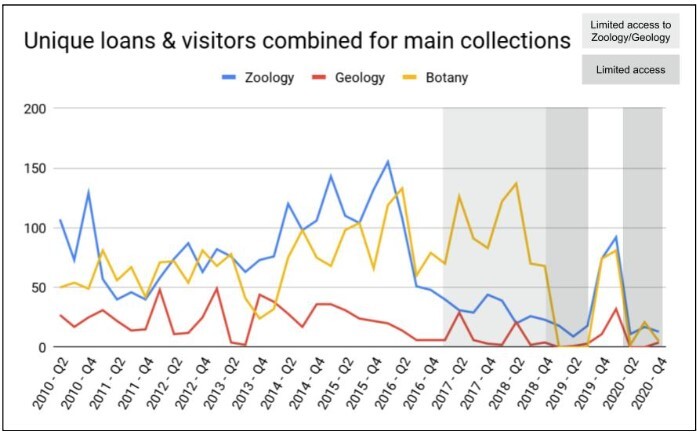
Quarterly total number of loans and visitors combined.

Fig. 2 shows the total number of quarterly visits and loans combined for a 10-year period between 2010 and 2020 for Botany, Geology and Zoology, the three major subcollections at Naturalis Biodiversity Center. Noticeable is that collection visits and loan requests do not occur in a steady flow, but fluctuate.

Besides the declines in the period 2017-2020 for the zoological and geological collections, caused by their closures or reduced access (see also Text box 1), Fig. 2 does not show a clear trend. A similar erratic trend is observed in the results for visits and loans separately. Fig. 4 shows unique loans sent out and the total number of specimens they contained for the period 1997-2020. We lack the data to statistically substantiate this, but overall it appears that both the number of loans and the number of loaned specimens have gradually decreased over the past decades. Continued measurements will show in the near future how the use of the collections by loans will recover after five years of disruption due to renovations and the pandemic.

Another aspect linked to use is the level of use in relation to other quantitative collection parameters, such as the overall size of collections, subcollections or other aspects like the presence of type material. Do collections that are larger in size or have a large number of type specimens, attract more visitors or loan requests? To look into such relationships, one has to compare collection-use between institutes. However, this only becomes possible and meaningful when collection use is recorded in a standardised way, based on well-defined parameters. Once information on use is widely shared between collection institutes, we can better understand factors affecting collection use. This will enable us as a community to make informed decisions to increase collection use and collaborate to optimise access for collection-based research. This paper also aims to start a discussion about generating standardised parameters for recording the scientific use of natural history collections. A first comparison between the level of use of different subcollections at Naturalis Biodiversity Center is presented below.

**Figure 3.**
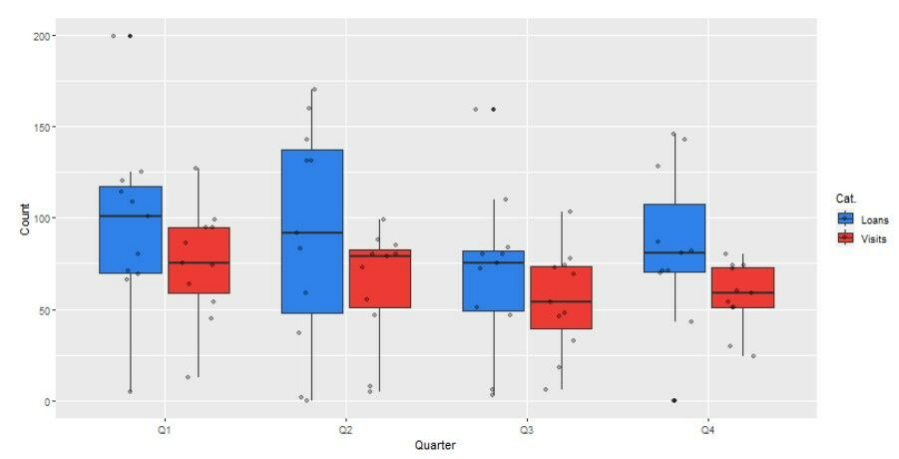
Quarterly number of loans and visitors combined.

In addition to overall patterns or trends over time, quantified data can also be used to detect yearly patterns. Reduced visits in specific quarters could be linked to the seasons or holidays. However, the data that are available for this study did not show any seasonal trends (Fig. 3).

**Comparison of geology, botany and zoology**

For the period between 2010-2020, Table 2 summarises the total number of unique visits and loans combined and the number of specimens included in loans per 1000 specimens for the geology, botany and zoology collections at Naturalis Biodiversity Center. Although being the smallest subcollection, botany received more visitors and sent out more specimens on loan than the zoology collection, which outnumbers the botany collection more than four times in size. The Geology collection, with roughly 1,5 times more specimens than the botanical collection, lags behind in visitors and loans and material sent on loan. Various factors cause these differences, such as the number of curators, their scientific networks, differences in access to the collections between 2010-2020 and the ease of preparing and sending out specimens on loans. Table 2 shows visits averaged per 1000 specimens as an index to compare collection use in the three subcollections. Such indexes can also be used to compare with use trends in other collections than those of Naturalis Biodiversity Center and can be helpful to make decisions and define strategies to optimise the use of collections.

At deeper taxonomic levels, for instance within the Vertebrate collection, there are also clear differences between various subcollections regarding the number of loans and visitors, as is shown in Fig. 5.

The Naturalis Biodiversity Center collections of geology, botany and zoology (Fig. 2), as well as the subcollections (Fig. 5) differ in many aspects and characteristics related to collection management, but also in aspects of physical use. It is easier to loan 100 small wasps than a single bird or a heavy rock specimen. Fig. 6 demonstrates that specimens that are more cumbersome to handle, like specimens from the Vertebrate or Geology collections, receive relatively more visits than loan requests, whereas collections that are small and easy to ship, like insects and invertebrates, receive relatively more loan requests. Note: not all loan requests are honoured. For instance, type specimens can only be studied at Naturalis Biodiversity Center and the same applies to historical collections (i.e. Von Siebold). Likewise large volumes of material are not lent out. In these cases, the researchers will have to visit the collection or, in the case of singular specimens, put in a digitisation-on-demand request to obtain digital images.

Besides differences in the ratio visits : loans per (sub)collection, one could also assess the relationship between overall collection size and number of specimens sent on loan at the subcollection level. In Fig. 7, the total number of specimens for the seven larger insect orders and the small insect orders combined sent on loan between 1984 and 2020 are plotted against the total number of specimens of the respective orders. A regression analysis showed that, for these groups of insects, there is a linear relationship that explains 84% of the variation between the number of specimens present in the collection and the total number of specimens sent on loan.

## Digital collection use

The analysis of digital use of the Naturalis collections covers the same time period as the physical collection use. The Naturalis Biodiversity Center has shared digitised specimen records with the Global Biodiversity Information Facility (GBIF.org) since 2010. By the end of 2020, the number of datasets had grown to 35, including one checklist dataset and 34 occurrence record datasets (Table 3). In total, the Naturalis Biodiversity Center mobilised 8,301,337 records to GBIF.org by the end of 2020. By then, 690,947 downloads were generated by users of the GBIF portal containing at least one specimen record from these datasets. This resulted in a total of 2,567 citations of individual datasets (Table 3) and contributions to 1,060 unique scientific journal articles. The Botany dataset with 4,972,211 records is by far the largest, from which records were included in at least 222,944 download events, resulting in 673 citations in scientific papers. The full list of scientific papers reporting citations to the Naturalis Biodiversity Center datasets can be found behind this link.

A linear relationship was detected when plotting the number of records against the number of downloads in log-log scale for each dataset that Naturalis Biodiversity Center shares with GBIF.org (Fig. 8). The following linear model was fitted:

10log(downloads) = 2,57106 +0,33308 x 10log(records)

downloads = 102,57106 x records 0,33308

p 0,001

R2adj. = 0,5601

The linear model explains 56% of the variation in the number of downloads for each dataset. When converted, the number of downloads is an exponential function of the number of records. Other factors that may influence the number of downloads for a dataset include date since publication, percentage of georeferenced records, geographical scope of the dataset etc. Nonetheless, a significant proportion of the variation in the number of downloads is not surprisingly explained by the number of records alone.

A cumulative plot of the number of records per year shows that the largest number of records was added in 2014 (Fig. 9- blue line). In that year, the ‘Naturalis Biodiversity Center (NL) - Botany’ was shared with GBIF.

GBIF started tracking downloads of data from 2013 onwards. Although the growth in the number of records shared with GBIF after 2014 was modest, the number of downloads from Naturalis datasets shows an exponential growth (Fig. 9 - red line). This shows that more and more researchers and policy-makers find and use GBIF-mediated data for scientific papers and policy decisions, a trend that continues today.

## Discussion

In this paper, an analysis is presented of the physical and digital use of the natural history collection of Naturalis. Despite the short timespan and the analysis being hampered by closures and moves, as well as alterations in the recording protocols, the results are used to discuss several aspects linked to the objective and transparent quantification of collection use, namely its relevance, its level of detail and completeness, digital collection use and its impact on physical use, but, first and foremost, standards to measure collection use.

**Standards**

Comparisons of collection use between institutes are cumbersome due to the lack of both standards for parameters as well as documents describing protocols. Obtaining transparent and objective data and information on collection use is not an end in itself, but is necessary to create reliable time series and meaningful comparisons between collections. The latter applies not only to individual institutes, but also to collections from different institutes. Global standards, let alone an index to quantify use of NHCs, are still lacking. No efforts have been made so far to develop international standards. Although this information is admittedly sensitive, the possibility of making meaningful comparisons about collection use and linking these to collection characteristics could provide a better understanding of the factors influencing use. This, in turn, could lead to the development of policies aimed at influencing or managing use and would certainly benefit the worldwide community of NHCs. A standard set of parameters is required to share information and to improve comparability of scientific use between NHCs. In order to prepare for a future where we can identify important patterns in the use of collections, a generic set of parameters is needed that can be applied to NHCs. Table 4 provides an overview of a generic set of parameters that play a role in collection use. We like to call upon TDWG, the body specialising in standards, as well as bodies aggregating information linked to collections, agents and specimens like GRSciColl, iDigBio or GBIF and those developing infrastructure like DiSSCo to make the development and implementation of standards describing collection use one of their priorities in the coming years.

**Relevance of standardised metrics on collection use**

Quantifying the use of collections in a standardised way allows us to objectively demonstrate the unique value of our collections to stakeholders. It documents trends and patterns, allows comparisons between collections and helps to evaluate decisions and policies on collection management. Detailed information on use provides valuable input for comparing, interpreting and evaluating the effect of certain factors on collection use, such as the number of taxa or type specimens present, the availability of laboratory facilities, the availability of a specialist or the logistics around travel and accommodation. Besides contributing to a long-term strategy, information about collection use could also be used to answer day-to-day questions. For instance, detailed analysis of use may assist collection managers in optimising the deployment of available (human) resources, to enhance access or direct digitisation policies. Gathering the right amount of data on use with the right level of accuracy and detail can be time-consuming, so it is essential to know for what you are gathering it. Is it a valuable tool for curation or does it only serve to inform management or external stakeholders? Over time, with the increasing automation of workflows and the digital availability of collection specimens, costs and efforts of gathering detailed data on the use of collections should decrease. International programmes such as DiSSCo or iDigBio promote the continuous digitisation of NHCs to disclose the vast amount of data available for biodiversity research, leading to initiatives such as the DiSSCo-UK Glossy to organise and finance digitisation efforts. The value of digitisation for research and curation has already been made clear (Popov et al. 2021), but it can also contribute towards developing a standardised approach to measure collection use across institutes worldwide.

**Detail and Completeness**

To a great extent, the level of detail in recording collection use determines the overall usefulness of the data and information gathered. Currently, the number of visitors, visitor days and loans, are parameters used to illustrate collection use at the collection (MfN Berlin; Smithsonian) or subcollection level (Tilley et al. 2024) as shown in the Naturalis Biodiversity Center example. Gathering information at lower hierarchical levels allows for a comparison of collection use at lower taxonomic or geographic levels. In addition, it also allows us to better assess differences in use between taxonomic groups and establish trends. Lastly, more detailed recording may help to start understanding relationships between collection characteristics (size, number of types) and collection use. When studying the available data on physical use in Naturalis Biodiversity Center, we found three shortcomings with regards to completeness of information. Firstly, collection managers play a key role not only in providing basic information to visitors about protocols (such as use of collection, species handling and loan requests), but also in registering visitors. However, it is quite challenging to ensure that registering is always carried out and not skipped due to lack of time, motivation or both. Secondly, the requests for visits, loans or digitisation that are not accepted - due to a variety of reasons - are not recorded. Thirdly, in addition to external visitors and loans, there are other categories of physical use that have not been quantified at Naturalis Biodiversity Center, such as use by internal research staff, information requests or small digitisation on demand requests. A fail-safe electronic way of recording a complete overview of the use that is being made as well as the requests that are unfortunately denied, would create an opportunity to better demonstrate the general demand for collections. This could also expose possible understaffing resulting in not utilising the collection’s full potential for societal and scientific use.

**Digital collection use and its impact on physical use**

**Figure 4.**
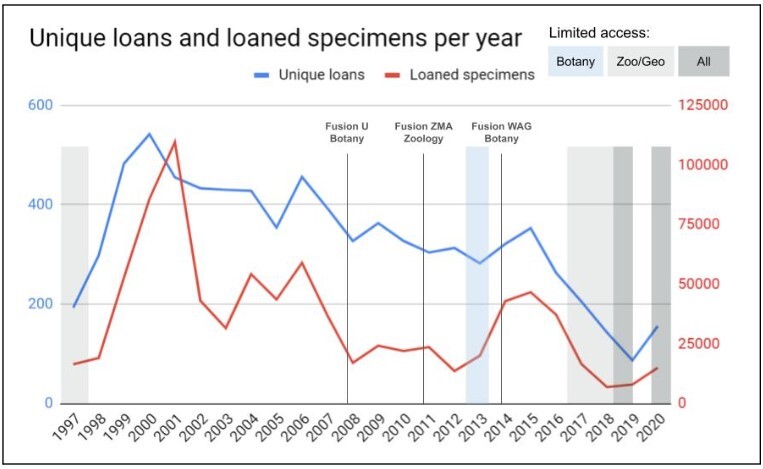
Number of outgoing loans (blue line) and specimens (red line) sent on loan per year.

**Figure 5.**
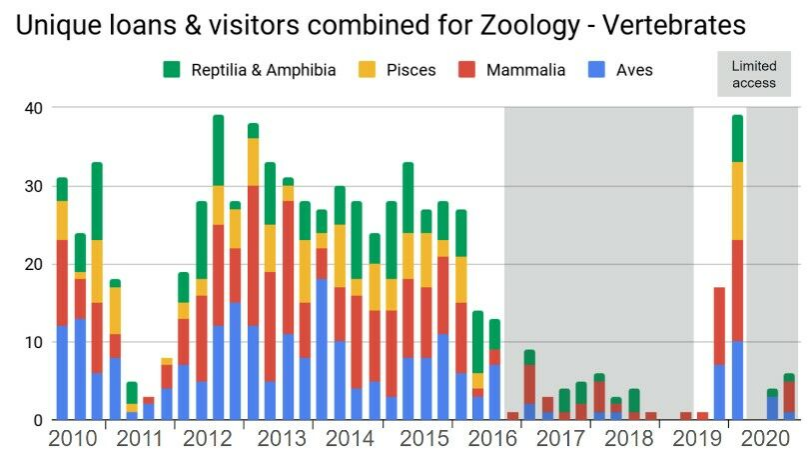
Quarterly number of Vertebrate loans and visitors combined for the taxonomic classes of Vertebrates.

**Figure 6.**
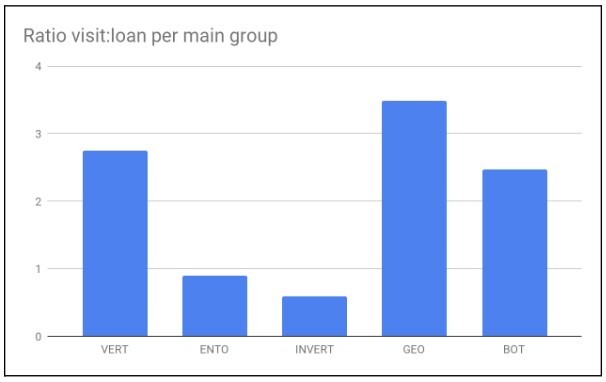
Ratio unique visits : unique loans for five different subcollections in the period 2010-2020. Vert: Vertebrates; Ento: Insects; Invert: Invertebrate excluding Insects; Geo: Geological collections; Bot: Botanical collections.

**Figure 7.**
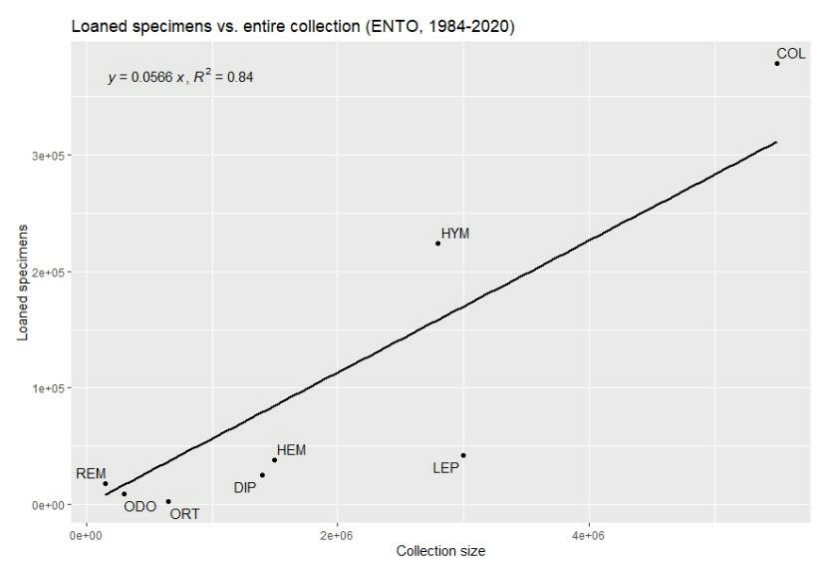
Total number of specimens per order(s) of insects sent on loan by Naturalis and its predecessors in the period 1984-2020 in comparison with the total number of specimens present in the collection for the respective order(s): COL: Coleoptera; DIP: Diptera; HEM: Hemiptera; HYM: Hymenoptera; LEP: Lepidoptera; ODO: Odonata; ORT: Orthopteroids; REM: remaining small insect orders.

**Figure 8.**
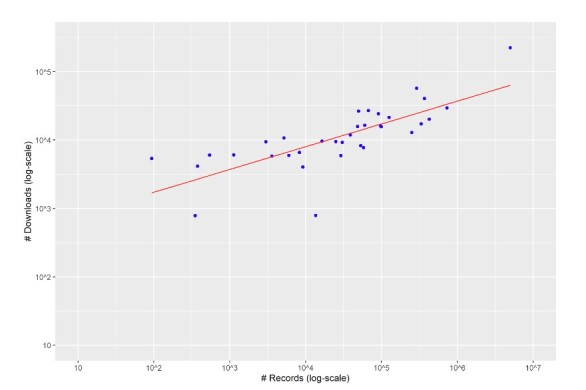
Number of records for the 34 occurrence record datasets of Naturalis Biodiversity Center plotted against the number of downloads of the respective dataset until 31/12/2020. Note the log-log scale of the graph.

**Figure 9.**
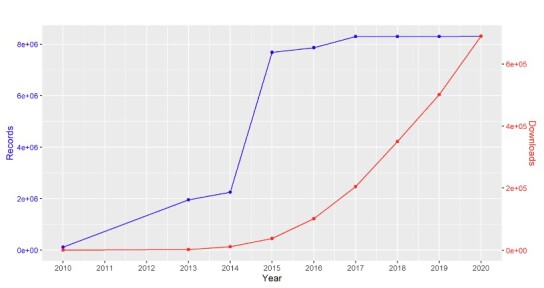
Cumulative number of records from Naturalis Biodiversity Center datasets at GBIF.org (blue line) and the cumulative number of download events from 2010 to 2020 (red line). Note different scales for the y-axes.

Besides taxonomy, digital specimen records contribute to many other scientific disciplines, including biodiversity informatics, macroecology, global change impact predictions etc. An analysis of the time period covered in this paper shows an exponential growth in the number of downloads of specimen-derived digital data from Naturalis (Fig. 9). Several researchers have investigated the impact of digitisation on the use of particular collections (e.g. Heberling et al. (2021)). At Naturalis, online publication of collection data did not result in reduced use of the physical collections of Naturalis (Fig. 4). With a proper international standardisation for gathering data on collection use, the impact of digitising collections can be further analysed. Understanding the changing patterns of use and the effects of digitisation will provide valuable insights in the impact of efforts, decisions and strategies we implement and offer policy-makers the tools to anticipate the future of collections-based research and proper curation of NHC. Can we distinguish trends of increased physical use due to increased online visibility or will some collections no longer need to be consulted physically because they are available online? For example, will loans become more targeted and, therefore, smaller due to prior online selection or larger because the researcher now knows what else is present in the collections?

The long-term preservation and proper management of NHC must be directed to optimise the use and usefulness of these facilities as an instrument for science. NHCs hold the physical evidence of our knowledge on biological diversity. NHCs are expensive facilities to maintain, with a long-term responsibility to keep them safe. To demonstrate the importance of this unique and globally distributed research infrastructure, it is important to continuously register the use and uses of NHCs and to do this in a transparent, objective and standardised way over time. If one considers curation of collections as a business case, the level of use being made of collections would be an inextricable part of the return on investment. With what level of use, if any, are we satisfied? Can we detect trends in collection use and identify the factors that positively or negatively influence these trends? Answers to these questions could be used to demonstrate and significantly increase the value of NHC. To answer them, the use of NHC needs to be quantified in a detailed, consistent and standardised way.

## Acknowledgements

During the COVID-pandemic, many museum guides of Naturalis have been indispensable in digitising and checking data from the historical loan administration and visitor log books. In this regard, particular thanks go out to Sarina Veldman, Mark Doeland and Frank Loggen. All Naturalis collection managers are gratefully acknowledged for providing information about physical use, historical documentation about use and the degree of digitisation of their collections, especially Roxali Bijmoer. Finally we are grateful for the advice of many senior staff and honourary researchers, such as Jan van Tol, Jan Wieringa and Gerard Thijsse, to better describe and interpret the historical data on use of the Naturalis collection.

## Figures and Tables

**Table 1.** Quantitative information about the Naturalis Biodiversity Center subcollections and their degree of specimen level digitisation.

Main Collection	Subcollection	# specimens	# specimens digitised
Botany	Vascular plants	5,230,000	4,897,500
Botany	Mosses	515,000	500,000
Botany	Algae	180,000	20,000
Botany	Fungi (incl. Lichens)	375,000	0
Zoology	Invertebrates - Arthropods	15,300,000	2,756,000
Zoology	Invertebrates - non-Arthropods	10,554,000	885,500
Zoology	Vertebrates: Fishes	200,000	98,000
Zoology	Vertebrates: Amphibians	50,000	52,500
Zoology	Vertebrates: Reptiles	50,000	
Zoology	Vertebrates: Birds	400,000	287,000
Zoology	Vertebrates: Mammals	100,000	77,500
Geology	Palaeontology: Botany & Mycology	100,000	64,000
Geology	Palaeontology: Zoology Invertebrates	3,000,000	
Geology	Palaeontology: Zoology Vertebrates	1,000,000	
Geology	Palaeontology: Other (other taxonomic groups)	4,671,000	
Geology	Mineralogy (e.g. rocks, ores, gems, minerals)	120,000	446,500
Geology	Other (e.g. fluid)	720,000	
Geology	Extraterrestrial: Collected on Earth (e.g. meteorites)	1,200	1,200
TOTAL:		42,566,200	10,085,700

**Table 2.** Index of unique visits and loans combined and the number of specimens sent on loan in the period 2010-2020 per one thousand specimens for the three main subcollections.

Naturalis Biodiversity Center subcollections and their size:	Geology (9,612,000)	Botany (6,300,000)	Zoology (26,654,000)

unique visits and loans / 1,000 specimens	0,08	0,44	0,11
specimens sent on loan / 1,000 specimens	6,36	32,06	21,48

**Table 3.** Datasets from the Naturalis Biodiversity Center that are available via GBIF.org and their statistics until 31/12/2020.

Dataset title	GBIF datasetKey	Date created	Records	Downloads	Citations
Naturalis National NHC (NL) – Invertebrate specimens from marine expeditions	fbe1f070-b6e4-11dd-81f6-b8a03c50a862	03/05/2010	30,436	9,259	38
Zoological Museum Amsterdam, University of Amsterdam (NL) - Bryozoa	b6ca95b0-c066-11dd-a312-b8a03c50a862	03/05/2010	3,004	9,449	31
Zoological Museum Amsterdam, University of Amsterdam (NL) - Diptera_Tipulidae_NL	1ed365f0-6167-11de-84c0-b8a03c50a862	03/05/2010	9,216	4,031	10
Zoological Museum Amsterdam, University of Amsterdam (NL) - Diptera_Tipulidae_Palearctic	b3a5b200-6167-11de-84c0-b8a03c50a862	03/05/2010	8,328	6,571	14
Zoological Museum Amsterdam, University of Amsterdam (NL) - Diptera_Types	ab0b73e0-c064-11dd-a311-b8a03c50a862	03/05/2010	5,194	10,712	29
Zoological Museum Amsterdam, University of Amsterdam (NL) - Invasive Insects	0cee5d00-6166-11de-84be-b8a03c50a862	03/05/2010	3,592	5,835	25
Zoological Museum Amsterdam, University of Amsterdam (NL) - Lepidoptera_Nymphalidae_Palearctic	562cf940-6166-11de-84bf-b8a03c50a862	03/05/2010	57,928	7,728	20
Zoological Museum Amsterdam, University of Amsterdam (NL) - Protozoa	f2836770-6166-11de-84bf-b8a03c50a862	03/05/2010	94	5,364	18
Naturalis Biodiversity Center (NL) - Coleoptera	843633a9-07cb-4918-a79f-6061b52c9dfd	09/01/2013	250,312	12,905	63
Naturalis Biodiversity Center (NL) - Lepidoptera	f3130a8a-4508-42b4-9737-fbda77748438	09/01/2013	427,788	20,129	100
Naturalis Biodiversity Center (NL) - Mollusca	d962a7dc-2183-4824-bb88-5e0ba14ec62d	09/01/2013	730,611	29,364	121
Naturalis Biodiversity Center (NL) - Hymenoptera	03f2256a-e548-43d7-a731-253302f4aa34	16/01/2013	333,214	17,217	69
Naturalis Biodiversity Center (NL) - Cainozoic Mollusca	a57e6526-54c6-465a-baa9-904979bbc93f	06/12/2013	99,021	15,685	75
Naturalis Biodiversity Center (NL) - Collembola	4f8de55f-5967-46c4-b689-31de17090ed4	26/03/2014	29,171	5,906	40
Naturalis Biodiversity Center (NL) - Amphibia and Reptilia	fccafd83-a934-4021-a112-4ae5fd39c14b	20/08/2014	49,732	26,438	86
Naturalis Biodiversity Center (NL) - Chelicerata and Myriapoda	b4aad9c3-518b-4550-82de-c70a7ead1b64	20/08/2014	91,47	24,252	124
Naturalis Biodiversity Center (NL) - Crustacea	0102d2af-7f21-4238-b1fa-cbbeeee60423	17/09/2014	125,82	21,467	114
Naturalis Biodiversity Center (NL) - Botany	15f819bd-6612-4447-854b-14d12ee1022d	16/09/2015	4,972,211	222,944	673
Naturalis Biodiversity Center (NL) - Cnidaria	4c7c21a8-68d3-49c5-b090-6b26259a291b	04/12/2015	60,347	16,404	94
Naturalis Biodiversity Center (NL) - Diptera	6a0a95c6-c07a-4c35-9e9f-f776e8730fd4	04/12/2015	53,428	8,262	49
Naturalis Biodiversity Center (NL) - Porifera	a4be4c6d-5ce7-47ba-982d-1deb05719133	04/12/2015	48,411	15,763	76
Naturalis Biodiversity Center (NL) - Aves	889c91a3-614f-4355-8df8-b6d0260a118c	07/12/2015	289,659	57,018	180
Checklist Dutch Species Register - Nederlands Soortenregister	4dd32523-a3a3-43b7-84df-4cda02f15cf7	30/09/2016			2
Naturalis Biodiversity Center (NL) - Brachiopoda	e66ff065-d823-4a4d-b129-569f649ba0eb	22/12/2016	1,133	6,074	23
Naturalis Biodiversity Center (NL) - Echinodermata	f598ca19-7c37-47e4-9a64-bcfe526847c1	22/12/2016	16,443	9,645	22
Naturalis Biodiversity Center (NL) - Foraminifera	1eb5e969-4412-4f08-81ec-3de057e559a1	22/12/2016	544	6,017	26
Naturalis Biodiversity Center (NL) - Hemiptera	8b42bbf1-b287-40a0-ab3a-88fb7928f8e2	22/12/2016	25,005	9,478	47
Naturalis Biodiversity Center (NL) - Odonata	306f61f2-9ff0-404e-8aa6-a525a7fae369	22/12/2016	38,842	11,898	28
Naturalis Biodiversity Center (NL) - Orthopteroids	5751c38c-078c-431a-be43-af27f5e38db2	22/12/2016	378	4,154	12
Naturalis Biodiversity Center (NL) - Pisces	19828488-7ebf-43f8-88af-6461d8afef9e	22/12/2016	97,619	15,962	45
Naturalis Biodiversity Center (NL) - Tunicata	9a025855-803d-4fa7-8417-ac7142544553	22/12/2016	6,006	5,95	15
Naturalis Biodiversity Center (NL) - Museum collection digitised at storage unit level	62d82928-dc6f-40dc-85b3-f2be47e7b49a	13/03/2017	368,853	40,574	238
Naturalis Biodiversity Center (NL) - Mammalia	009a76f6-0960-4a56-a116-63991e6bb037	03/08/2017	67,177	26,92	59
Aspilanta new genus (Heliozelidae) specimen data	db2db3cd-5473-43be-b57a-95eff336f09c	11/06/2020	350	782	1
Total			8,301,337	690,157	2,567

**Table 4.** Proposed parameters for scientific use of collections.

	Use	Category	Parameters	Resolution - Frequency
1	Physical	Loans	purpose, scope, amount, duration	hierarchical taxon level; period
2	Physical	Visitors	purpose, scope, amount, duration	hierarchical taxon level; period
3	Physical	Internal staff use	purpose, scope, amount, duration	hierarchical taxon level; period
4	Physical	Digitisation on Demand	purpose, scope, amount, duration	hierarchical taxon level; period
5	Physical	DNA sampling	purpose, scope, amount	hierarchical taxon level; period
6	Digital	Downloads	purpose, scope, amount	hierarchical taxon level; period
7	Digital	Specimen citations	purpose, scope, amount	hierarchical taxon level; period
